# Picomolar Detection of Hydrogen Peroxide using Enzyme-free Inorganic Nanoparticle-based Sensor

**DOI:** 10.1038/s41598-017-01356-5

**Published:** 2017-05-02

**Authors:** Craig J. Neal, Ankur Gupta, Swetha Barkam, Shashank Saraf, Soumen Das, Hyoung J. Cho, Sudipta Seal

**Affiliations:** 1 Advanced Materials Processing and Analysis Center, Materials Science & Engineering, University of Central Florida, 4000 Central Florida Blvd, Orlando, FL 32816 USA; 2 Nanoscience Technology Center, University of Central Florida, 4000 Central Florida Blvd, Orlando, FL 32816 USA; 3 Mechanical & Aerospace Engineering, University of Central Florida, 4000 Central Florida Blvd, Orlando, FL 32816 USA; 4 College of Medicine, University of Central Florida, 4000 Central Florida Blvd, Orlando, FL 32816 USA

## Abstract

A philosophical shift has occurred in the field of biomedical sciences from treatment of late-stage disease symptoms to early detection and prevention. Ceria nanoparticles (CNPs) have been demonstrated to neutralize free radical chemical species associated with many life-threatening disease states such as cancers and neurodegenerative diseases by undergoing redox changes (Ce^3+^  ↔ Ce^4+^). Herein, we investigate the electrochemical response of multi-valent CNPs in presence of hydrogen peroxide and demonstrate an enzyme-free CNP-based biosensor capable of ultra-low (limit of quantitation: 0.1 pM) detection. Several preparations of CNPs with varying Ce^3+^:Ce^4+^ are produced and are analyzed by electrochemical methods. We find that an increasing magnitude of response in cyclic voltammetry and chronoamperometry correlates with increasing Ce^4+^ relative to Ce^3+^ and utilize this finding in the design of the sensor platform. The sensor retains sensitivity across a range of pH’s and temperatures, wherein enzyme-based sensors will not function, and in blood serum: reflecting selectivity and robustness as a potential implantable biomedical device.

## Introduction

As new therapies and technology are developed, the medical community is seeing a change from late-stage disease treatment to early detection and prevention^1^. This way, treatments are becoming pro-active, rather than reactive, decreasing the incidence or severity of serious and chronic illnesses. This approach has seen great success for those afflicted with early signs of cancer or neurodegenerative diseases such as Alzheimer’s, Parkinson’s, and multiple sclerosis^2–7^. Common to all of these ailments are the production of reactive oxygen species. Specifically, reactive oxygen species evolve as a result of altered, cellular metabolism arising from a given disease state. These species are highly unstable and induce redox of cell structures leading to activation of immune response and apoptosis. Among these hydrogen peroxide (H_2_O_2_) has been well-studied as an analyte to describe disease condition.

H_2_O_2_ detection is critical to manufacturing, food production, pharmaceuticals, and medicine. Specifically, precise H_2_O_2_ detection and quantification is a necessity in food sterilization processes, pharmaceuticals production, and medical devices. Detection has been accomplished using a number of different techniques; namely: titration^8^, spectroscopy^9^, fluorescence^10^, chemiluminescence^11^, and electrochemical methods. Of these methods, electrochemistry is arguably the simplest: producing fast and precise data while requiring only limited instrumentation, and can be accomplished through analyte oxidation or reduction. Further, detection can be accomplished via simple voltammetric, impedance, and/or amperometric methods. These methods can also be coupled with optical techniques to produce even greater levels of precision (i.e. electrochemical luminescence and photoelectrochemical methods)^12, 13^. In the past, the technique’s main limitation has been the large overpotential required to induce redox reactions and slow electron transfer kinetics^14^. Recently, these shortcomings have been overcome through the use of modified electrodes. Specifically, electrodes have been modified with small redox-active molecules, polymers, enzymes, and nanomaterials^14, 15^. The simplicity and versatility of these electrochemical sensors, strongly suggests their use in biosensors.

Within the field of sensor technology, devices for bio-sensing have seen an especially pronounced growth. Among these, enzymes are used most often for electrochemical sensors and especially in biosensors. Enzyme sensing elements have high sensitivity, selectivity, and fast time of response: making them well-suited for biosensors. However, their function is limited to specific solution conditions and variance from these conditions in pH, ionic strength, temperature, or light exposure can result in significant, in some cases irreversible loss of activity. In the case of H_2_O_2_ detection, the most commonly used enzyme is horseradish peroxidase (HRP)^16–18^. However, this protein loses activity as pH changes from pH 8 to 4 (−>60% of initial activity) and from 40 to 20 °C (−30%) when immobilized^19^. This loss in activity has been attributed to changes in tertiary structure, which is lost completely at 42 °C^20^. In developing more robust sensors, researchers have turned towards enzyme-free platforms^21–23^. Often, this is accomplished by incorporation of inorganic nanoparticles (e.g. platinum, carbon nanotubes, palladium, iron oxide)^21–23^. Use of these materials have produced sensors with comparable sensitivities to enzyme-based sensors while lifting the restriction to mild conditions. Among these, cerium oxide (ceria) has demonstrated substantial ability to interact with and allow detection of significant analytes^24, 25^.

Ceria has demonstrated wide, biomimetic reactivity towards reactive oxygen and nitrogen species^25–29^. This enzyme-mimetic behavior is related to the ratio of Ce^3+^ to Ce^4+^ with higher or lower ratios being better suited for different chemical substrates. Additionally, as the dimensions of the ceria lattice are decreased to the nano-scale, the prevalence of Ce^3+^ increases along with the number of oxygen vacancies due to an increase in bond strain^30^. Therefore, ceria nanomaterials show unique activity and reactivity^28, 31–33^. Thereby, experimental use of ceria nanomaterials as therapeutic agents for cancers, neurodegenerative diseases, and ophthalmological diseases has been highly successful^34–43^. Experimental use of ceria has allowed neutralization of reactive oxygen species and an increase in overall cell viability. In particular ceria interaction with H_2_O_2_ has been especially well-studied.

Several studies have been produced which use ceria nanoparticles (CNPs) as sensing elements for H_2_O_2_^24, 44–46^. However, these studies do not demonstrate high sensitivities. Further, the use of CNP-based sensors for biomedical applications have not been studied in blood (protein-containing) conditions, for evaluation of the platform as an implantable, long term use device. In this study, the electrochemical response of CNPs in presence of H_2_O_2_, chosen as a surrogate molecule to determine concentrations of reactive oxygen species, is characterized. We use different preparations of CNPs to determine whether the ratio of Ce^3+^:Ce^4+^ has a direct relationship with the electrochemical response. Then, the determined relationship is used to develop a biosensor platform optimized for electrochemical response to ultra-low concentrations of H_2_O_2_ and functionalized to disallow protein adsorption on the particle surface. This sensor is then tested in blood serum to evaluate the sensors performance in presence of potentially surface-fouling proteins, mimicking the environment experienced by biomedical devices.

## Results and Discussion

### CNP Physicochemical Characterization

For this study, three different ceria nanoparticle (CNP) formulations (CNP1–3) were produced by varied synthesis methods. X-ray photoelectron spectroscopy (XPS) was performed for each of these to confirm differences in Ce^3+^:Ce^4+^ ratio (Table 1). This ratio was found to decrease across the formulations from CNP1 → 3. Before comparing formulations of varied Ce^3+^:Ce^4+^ for electrochemical activity, it was necessary to characterize certain physicochemical properties for each; namely: particle size, morphology, and zeta potential (collected in Table 1).

**Table 1:** Ceria Nanoparticle properties.

	Morphology	Size (nm)	Zeta Pot. (mV)	Ce^3+^:Ce^4+^ (from XPS)
CNP1	Spherical	3–5	4.571	1.06
CNP2	Spherical	3–5	3.245	0.39
CNP3	Spherical	3–5	1.336	0.24

**Figure 1:**
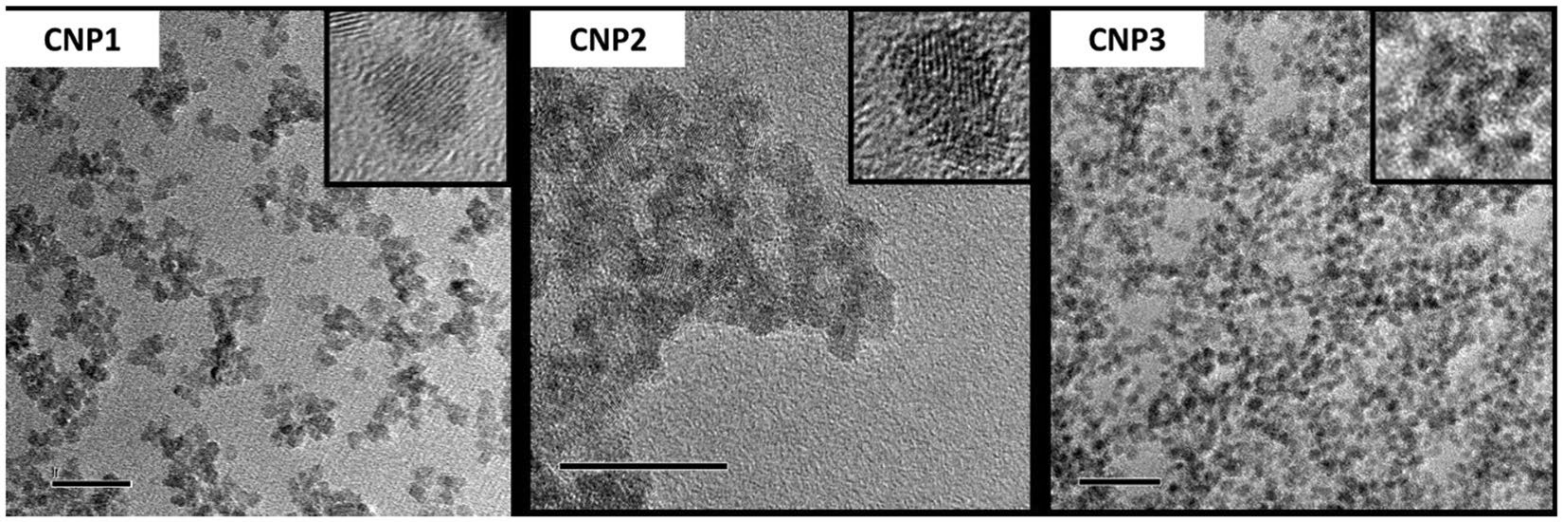
CNP (Left to Right, CNP1,2,3) size characterization (TEM images) with insets illustrating crystallinity (all scale bars are 20 nm). All formulations show aggregation due to the high reactivity of NP surfaces. Images show well-formed, individual crystallites. Further, these images highlight the crystallinity of these particles through the clear presence of lattice fringes.

Particle size and morphology were similar for all formulations. Transmission electron microscopy (TEM) micrographs (Fig. 1) show spherical, ~5 nm CNPs. Based on these images and the properties collected in Table 1, we determine that the formulations show particles of comparable size, morphology, and zeta potential. From here, the ability of each formulation to chemically degrade H_2_O_2_ was determined via catalase assay.

**Figure 2:**
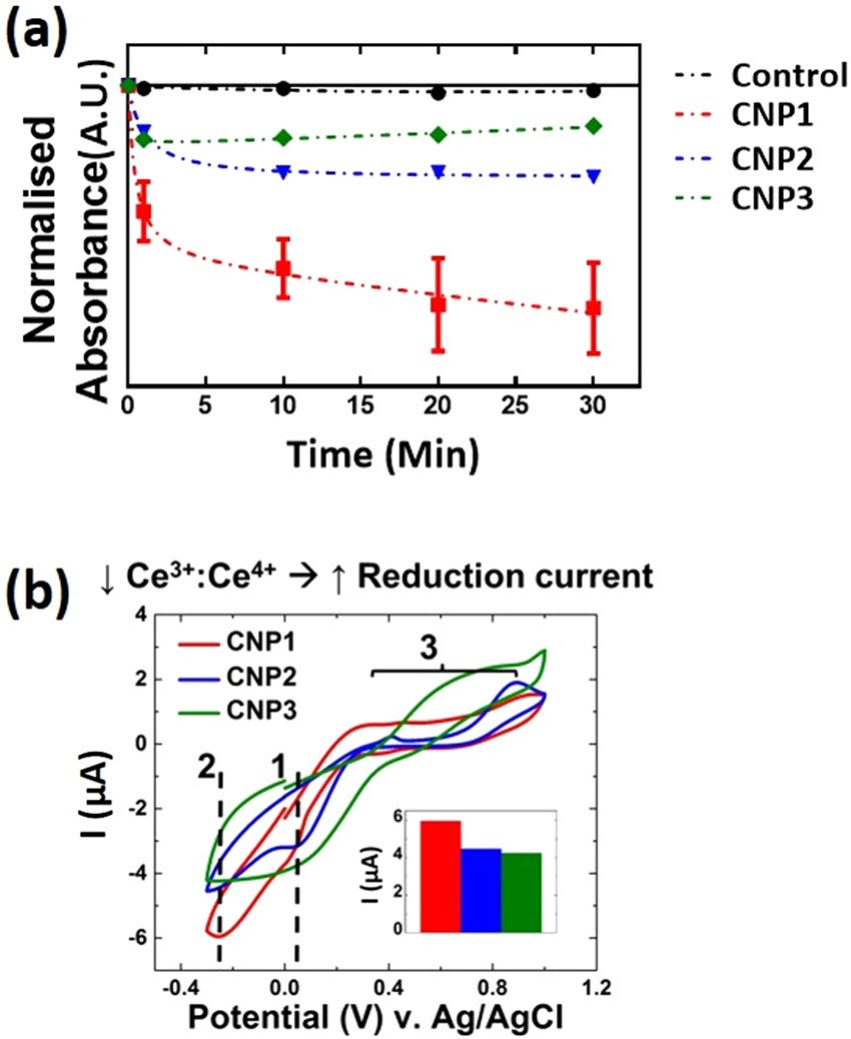
(**a**) Catalase assay. Activity was greatest for CNP1 followed in descending order of activity by CNP2 and 3. **(b)** CV for all CNP preparations (1 mM H_2_O_2_ and CNPs), cathodic peak current (inset). The inverse relationship between Ce^3+^:Ce^4+^ ratio and catalase activity holds for CV. Additionally, an anodic peak present for all formulations shows a positive relationship between Ce^3+^:Ce^4+^ ratio and current.

Catalase assays were performed for all CNP formulations (Fig. 2a) and it was found that lower Ce^3+^:Ce^4+^ ratios produced greater catalase activity (i.e. activity increased from CNP1 → 3) due to the redox conversion of Ce^4+^ → Ce^3+^, corroborating the findings of past studies^[2a]^. Given that particles from each formulation varied only in Ce^3+^:Ce^4+^ ratio and that catalase activity was found to vary with this ratio, we next performed electrochemical studies to evaluate whether current response showed a similar relationship.

### CNPs Electrochemical Characterization

Initially, to characterize the electrochemical behavior of each CNP formulation, CV was performed with 1 mM CNPs and 1 mM H_2_O_2_ for all formulations (Fig. 2b). CV scans were completely consistent by the third cycle (used for analysis), with subsequent cycles being largely similar (data not shown). Since the current does not change considerably with successive cycles, we determine that the observed current response does not arise from any side reactions in the solutions (i.e. not from an ECE-type mechanism wherein an electrochemical change elicits a chemical reaction mediating a current signal)^19^. The overall shape of the obtained CV curves were fairly similar across formulations, suggesting similar electrochemical reactions for all formulations^47^. The peaks common to all formulations occur at ~0 V (hereafter, *peak 1*), −0.23 V (*peak 2*) and 0.4–0.7 V (*peak 3*). These are attributed to the CNP-mediated reduction of H_2_O_2_ (*peaks 1 and 2*) and the oxidation of the Au electrode (*peak 3*)^48, 49^, respectively.

CNPs with lower Ce^3+^:Ce^4+^ ratio have a more defined peak 1; this has been attributed to the initial reduction of H_2_O_2_^48^. In contrast, the higher ratio CNP1 solution shows a more diffuse, wide peak at a similar potential; this behavior is consistent with complex catalysis mechanisms presented in literature^49^. Specifically, the peak character suggests that reduction initially occurs, however, the catalysis is kinetically restricted for high Ce^3+^:Ce^4+^ formulations. Degradation of H_2_O_2_, for these formulations occurs very slowly, on the order of days for millimolar concentrations in absence of applied electrical potential^50^. Additionally, it has been speculated that this diffuse peak represents a multi-step reduction mechanism wherein radical oxygen species (such as superoxide) and/or oxygen are evolved and transiently adsorb at the electrode surface^21^. Peak 2 represents the complete reduction of H_2_O_2_. This peak is seen to be sharp and intense for lower Ce^3+^:Ce^4+^ ratio CNP formulations indicative of a fast, diffusion-limited reduction while higher ratio formulations show a more diffuse peak which seems to overlap with peak 1 (as detailed above). In similar work^13, 49^, this cathodic peak was attributed to the CNP-mediated reduction of surface-complexed O_2_^2−^/O_2_^−^ species. The high current increase seen for low ratio formulations was attributed to peroxy-complexed CNPs functioning as nano-electrodes by colliding with the working electrode and facilitating indirect electron transfers from the working electrode to adsorbed oxygen species^49, 51, 52^. Particle contact with the conductive electrode resulting in degradation of the surface species occurs primarily through the following relationship,

1 $${{\rm{Ce}}}^{4+}{:H}_{2}{{\rm{O}}}_{2}\to {{\rm{Ce}}}^{3+}+{{\rm{H}}}_{2}{\rm{O}}+{{\rm{O}}}_{2}.$$

The peak shape/character arises from the high surface coverage of the nanoparticles by the peroxy species: allowing reduction of a large quantity of molecules near-simultaneously^49^. In support of this, in a study utilizing CNP collision with microelectrodes, individual CNP impacts were observable spikes in current observed for peroxide adsorbed particles relative to CNPs without H_2_O_2_ exposure^49^.

Performing CVs at multiple scan rates and plotting the square root of each scan rate vs. the corresponding peak current (Supplementary Figure S2), we determined that the CNP-mediated peroxide reduction reaction is diffusion-limited^47^. It is important to note that the reduction response is produced by nanoparticle flux at the electrode surface, the current signal is dependent on the diffusivity of the nanoparticles, rather than of the peroxide species.

In agreement with catalase data, we observed that lower Ce^3+^:Ce^4+^ ratio formulations produced a nearly two-fold greater cathodic peak current density as compared to higher ratio formulations (determined by normalizing peak currents with respect to electrode electroactive surface area, described in the Experimental section). Figure 2b details the relative peak current densities for each of the formulations. Lower Ce^3+^:Ce^4+^ ratio better facilitate H_2_O_2_ reduction based on their oxygen release property. Oxygen release from the CNP lattice is believed to be more favorable for CNPs with higher Ce^4+^ than is for higher Ce^3+^ particles^53^. This may allow for a more favorable reduction reaction by releasing reaction products at a higher rate: explaining the greater cathodic current density for CNP3 over CNP1 as well as the overall shape of the respective CV curves.

Additionally, we saw the reverse trend for anodic current density with higher ratio formulations at peak 3 producing greater current density, similar to literature SOD activity trends^25^. This peak was attributed to Au oxidation and has been shown to be sensitive to H_2_O_2_ concentrations (Supplementary Figure S3)^48^. However, no consistent trend with H_2_O_2_ concentration was observed for this signal and therefore the signal could not be used for analytical measurements (Supplementary Figure S3). From the present study, we cannot determine conclusively whether the observed oxidation reaction (Peak 3) can be ascribed to CNP SOD activity.

Due to the strong relationship between Ce^3+^:Ce^4+^ ratio and cathodic current, we determined to study the CNP-mediated H_2_O_2_ reduction current response. CA is more suited for quantitative measurements, such as these, over CV^47^. Therefore, the formulations with the least (CNP1) and greatest (CNP3) Ce^3+^:Ce^4+^ ratio were selected for further investigations to confirm the trends in current values observed in CV and to test the efficacy of the particles as an amperometric assay.

**Figure 3:**
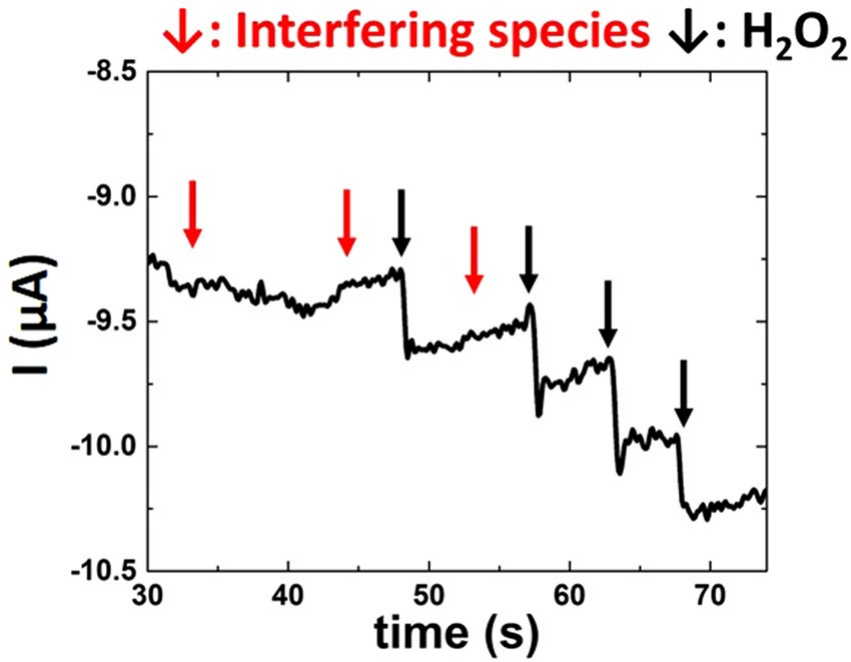
CNP3 CA with addition of interfering ﻿s﻿pecies and H_2_O_2_. Interfering species addition (200 μM uric acid, glucose, or nitrite) causes no significant change in current. H_2_O_2_ addition causes immediate response with each addition.

CA was performed with several interfering species (chemical substances which experience redox at un-modified electrode surfaces within the tested potential range, are redox agents that could react with ceria, or could react with catalysis products and alter measurements), shown elsewhere to poison H_2_O_2_ redox reactions, and with successive additions of 200 μM H_2_O_2_ to CNP1 and 3 formulations in dH_2_O (Supplementary Figure S4)^44^. None of glucose, uric acid, nor sodium nitrite produced any significant current change (Fig. 3) suggesting strong selectivity towards the target analyte. Further, addition of interfering species and H_2_O_2_ did not produce any measurable response in absence of CNPs (data not shown). However, addition of ascorbate produces a chemical reaction with ceria and elicits a weak electrochemical response (Supplementary Discussion S4)^54^. This interaction is addressed, later, for the formation of a biosensor. Upon addition of H_2_O_2_ to CNP solutions, an immediate current response was observed (Fig. 3). Comparing CA data for CNP1 to CNP3 formulations shows significantly greater (>125%) current values for CNP3, similar to the differences observed in CV measures (Supplementary Figure S4). In comparison with CV data, the increased current response likely arises from the proximity of CNPs to the electrode surface. Particle-mediated H_2_O_2_ reduction is limited by H_2_O_2_ diffusion to the particle-coated electrode surface, rather than by the CNP diffusion limitation for the solution-based CNP method. Therefore, the electrode experiences a greater flux at its surface immediately following peroxide addition, increasing the achieved signal.

Based on these results we believe that CNP formulations with lower Ce^3+^:Ce^4+^ are better suited for amperometric detection of H_2_O_2_. Further, due to the success of the CNP3 formulation in producing a considerable current, we fabricated a sensor by immobilizing CNPs on a glassy carbon electrode. To optimize our sensor design and in considering the generally observed trend of increasing ionic diffusion for nanomaterials over micron-scale materials, we sought to characterize the influence of nano-scale chemical properties of the ceria film^55, 56^.

**Figure 4:**
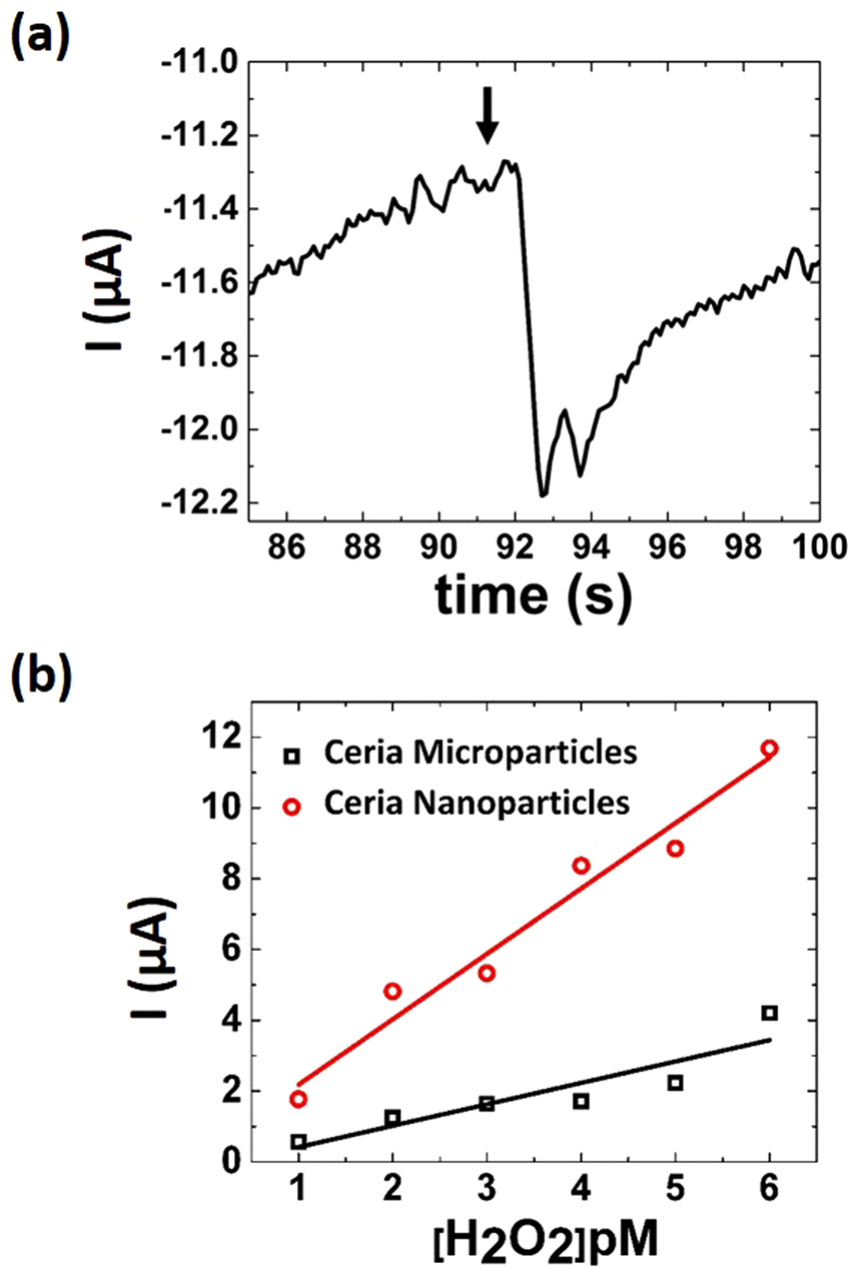
(**a**) Picomolar detection by biosensor. Addition of H_2_O_2_ to a final concentration of 1 pM produces a 350 nA signal, compared with the nanomolar limit of detection of most enzymes. **(b)** Nano v. Micro particle characterization. CNPs show significantly greater sensitivity towards H_2_O_2_ analyte as compared to ceria microparticles-based sensor; this greater signal is attributed to nano-size effects and specifically to the change in nanomaterial surface chemistry/physics.

To accomplish this, measurements from the sensor described above were compared with those of an analogous sensor based on a ceria microparticle film (Fig. 4b). As shown, the CNP-based sensor boasts a significantly greater sensitivity. Ostensibly, the nano- character of the particles allows for more efficient/effective surface catalysis resulting in a greater electrochemical signal. This character has been well-studied in ceria and is often ascribed to the bond strain produced in ceria when reduced to nano-dimensions (increasing concentration of Ce^3+^ at surface)^57^. Therefore, the CNP-based sensor platform was further analyzed for detection ability.

**Figure 5:**
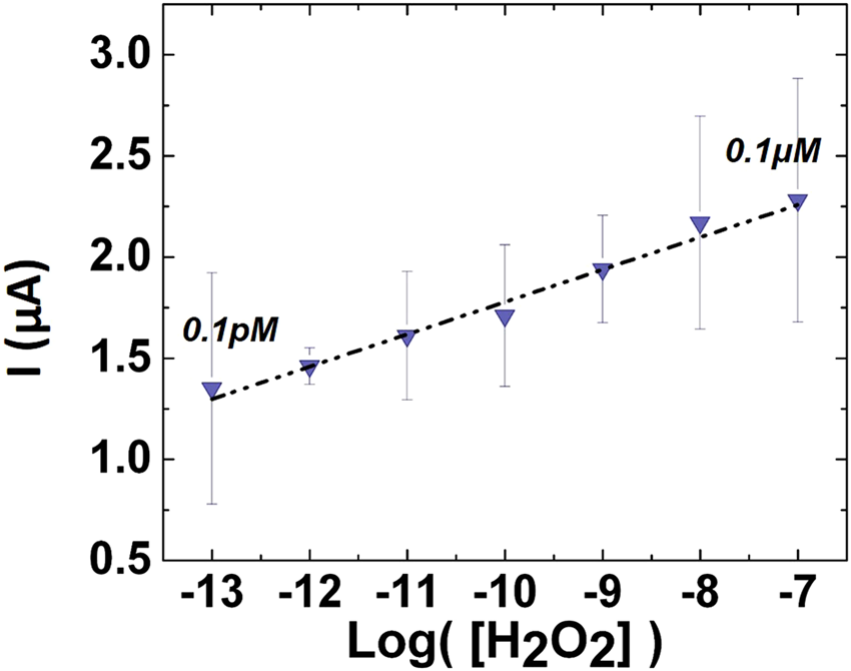
Sensor detection range for H_2_O_2_. The detection range is across seven orders of magnitude from 0.1 pM to 0.1 μM. The concentration is represented as the log value of the molar concentration. Sensitivity is determined from semi-log plot as: 0.156 μA/log(M)*cm^2^.

CA was performed to determine the H_2_O_2_ detection range for the CNP-based device. The sensor was able to produce signals at the picomolar (parts per trillion) concentration level (Fig. 4a). This level of detection is highly competitive with enzyme-based sensors which typically function in the nanomolar region^45^. Further, the sensor is capable of detecting concentrations orders of magnitude lower than comparable inorganic material-based sensors (Table 2). It was found that the sensor produced reliable signals across seven orders of magnitude (from 0.1 pM to 0.1 μM) (Fig. 5). The lower bound of this range was determined using the convention for limit of quantitation (LOQ); specifically,

$${\rm{LOQ}}=({\rm{Root}}\,{\rm{Mean}}\,{\rm{Square}}\,{\rm{of}}\,{\rm{Blank}}\,{\rm{current}})+10\,{\sigma }_{{\rm{RMS}},{\rm{Blank}}}$$

**Table 2:** H_2_O_2_ Sensors and their Properties.

Detection Element	Linear Range	Sensitivity	LOD	Ref.
CNP	0.1 pM–0.1 μM	0.156 μA/log(M)*cm^2^		(This Work)
Prussian Blue deposited on GC	200 nM–0.15 mM	0.6 A/M* cm^2^	0.8 nM	61
Benzylamine stabilized AgNPs	100 μM–100mM		31.3 μM	62
CuO Nanoflowers	42.5 μM–40mM	88.4 A/mM*cm^2^	0.167 M	63
Mesoporous Pt electrodes from LCs	20 μM–40 mM	2.8 A/mM*cm^2^	4.5 μM	64
AgNPs on GC	5–50 μM			65
CoFe_2_O_4_ magnetic NPs in beta-cyclodextrin	0.1–4 μM		2 μM	66
Poly(pyrrole)-Ni:hexacynoferrate			0.2 nM	67
Mg-modified Si nanowire thin film	‘Up to 10 mM’	78.9 (+/−) 2.9 nA/mM	0.1 mM	68
HRP micro-encapsulated in tetraethyl orthosilicate + luminol	100 μM–3 mM		0.67mM	69
Ag microspheres			1.2 μM	70
Catalase at amine-functionalized graphene coated AuNPs	300 nM–600 μM	13.4μA/mM	50 nM	71
Electrodeposited AgNPs on collagen I modified GC	5 μM–40.6 mM		0.7 μM	72

The obtained range includes concentrations relevant to physiological conditions. Beyond this, the LOQ of this detection range is several orders of magnitude lower than that of enzyme-based devices (nanomolar limits). Further, in comparing with other enzyme-free H_2_O_2_ sensors, we see that the tested platform has a limit of quantitation 3 orders of magnitude lower than any of the reviewed sensors; with a comparable range of detection (Table 2). The sensors sensitivity was determined from a semi-log plot of current v. log([H_2_O_2_]) (Current = (sensitivity/cm^2^)(log [H_2_O_2_]) + (constant)) to be 0.156 μA/log(M)*cm^2^. Additionally, chronoamperometric measurements evidence a time of response of ~6 seconds. From here, the sensor platform was tested for robustness in different chemical environments.

**Figure 6:**
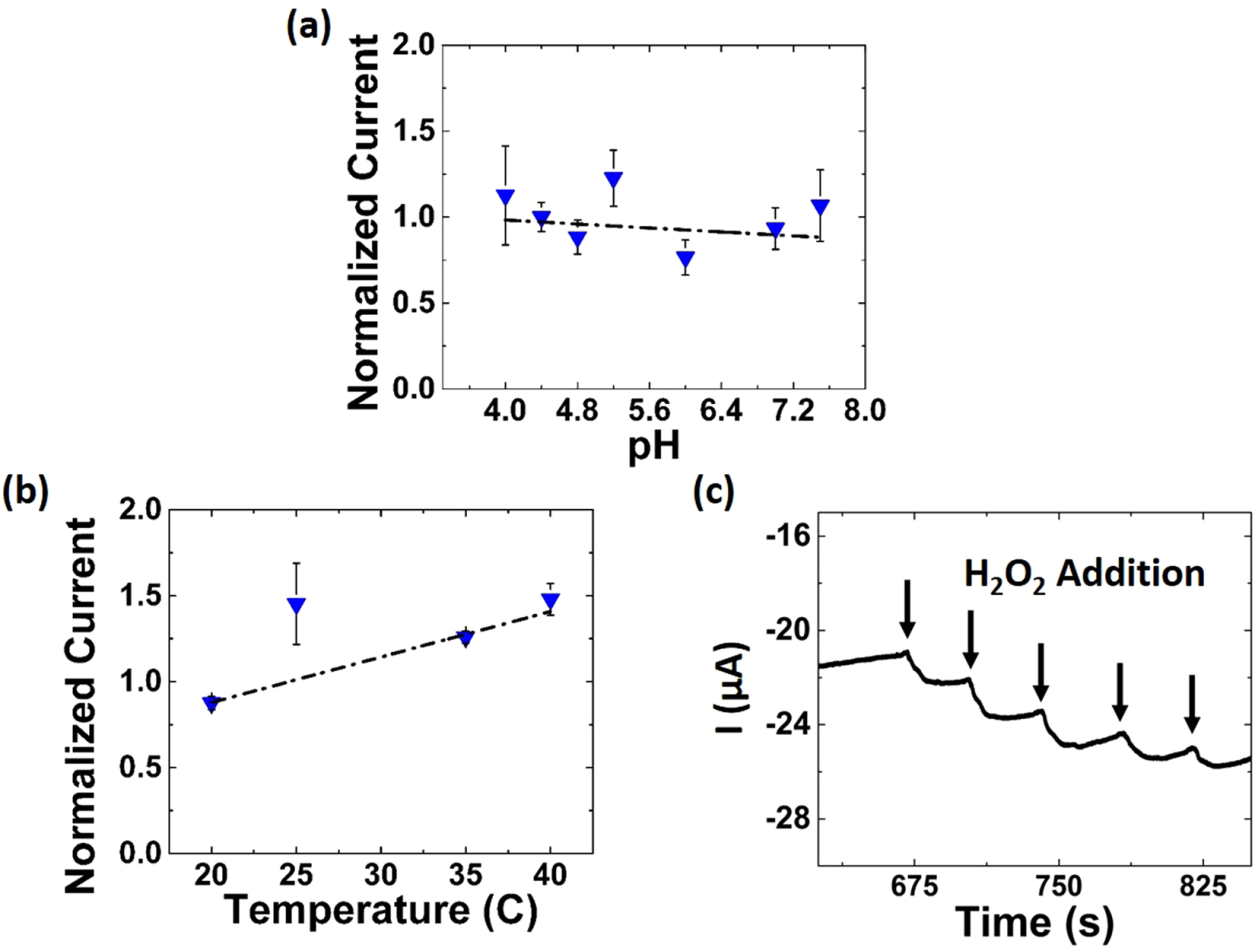
Sensing in harsh bio-conditions. The sensor shows no loss in activity from **(a)** pH 4.0 to 7.6 (st.dev: 27.3%) or from **(b)** 20 to 40 °C (st.dev: <5%), as compared with HRP which loses significant activity across these ranges. Further, experiences a melting point at 42 °C resulting in loss of tertiary structure. **(c)** CA with Nafion-coated CNP-based sensor in blood serum. Serial additions of 1 μM H_2_O_2_ produces clear current signal suggesting absence of bio-fouling. Sensitivty in blood serum, after surface functionalization: 0.103 μA/log(M)*cm^2^.

Figure 6a,b shows that the sensor experiences no loss in activity across the measured pH (4–8) (standard deviation: <5%) and a slight thermal activation across temperature range (20–40 °C) (standard deviation: 27.3%). Further, as a ceramic material, CNPs exhibit high thermal stability with structure changes only occurring well beyond temperatures practical for biosensing^58^. In order to utilize the sensor platform as a true biosensor it must be stable against solution leaching and biofouling. The as-synthesized glassy carbon electrodes have a moderately porous structure (Supplementary Figure S6). We believe that this structure played an important role in maintaining CNP-adsorption on the electrode surface as previous attempts with gold working electrodes used for solution-based CNP electrochemical characterization saw significant CNP layer leeching into test solutions (data not shown). However, the CNP comprised film is still vulnerable to degradation and in bio-solutions the surface can be blocked by adsorbed proteins (bio-fouled)^59, 60^. In addition, sensor operation could be disturbed solely by the change in solution electrochemical properties (due to proteins). Therefore, we next applied a thin layer of Nafion to the sensor and tested in blood serum^23^.

CA measurement in blood serum showed clear current response upon addition of analyte (Fig. 6c) whereas measurements using sensors without Nafion produced no signal. The sensor retained a sensitivity of 0.103 μA/log(M)*cm^2^. Further, incubation of the sensor in serum overnight caused no additional loss in sensitivity (data not shown) suggesting resistance to bio-fouling at short time scales. In the future, the stability of the sensor in bio-fouling solutions will be analysed to assess its potential utility as an implantable sensor. Additionally, the Nafion layer prevented the non-specific interaction between ceria and ascorbate. Thereby, the interference from these species was eliminated: further recommending this device platform as a clinical biosensor.

## Experimental Section

### Materials

Nano-pure diamond distilled water; 30% NH_4_OH, 30% Hydrogen Peroxide, Cerium nitrate hexa-hydrate (trace metals basis, 99.999%), 1% Nafion, poly(acrylonitrile), sodium phosphate dihydrate, sodium diphosphate monohydrate, sodium acetate, hydrogen chloride, and sheep’s blood serum from Sigma Aldrich; Cerium Oxide nanoparticles 20% colloidal suspension in water from Alfa-Aesar; powder cerium oxide microparticles from REaction a John Mathis company.

### Particle Synthesis and Characterization

For this study, three different formulations were produced which were demonstrated in other studies to have dissimilar Ce^3+^:Ce^4+^ ratios.

CNP1: Synthesized by dissolving cerium nitrate hexahydrate in 20 mL of dH_2_O followed by addition of a stoichiometric amount of 30% H_2_O_2_. CNP2: Alfa-Aesar CNPs were diluted using dH_2_O, as required for given measurements. No other processing procedure was performed on these particles. CNP3: NH_4_OH precipitated CNPs were produced through hydrolysis. Then, this solution was centrifuged at 8000 rpm to collect precipitated particles. The collected particles were then re-suspended in dH_2_O and titrated to a pH of ~4 using nitric acid.

All CNPs were characterized using XPS (Physical Electronics (PHI5400 ESCA) spectrometer with a monochromatic Al Kα X-ray source at 300 W with a base pressure of 5 × 10^−8^) Torr) to determine the Ce^3+^:Ce^4+^ ratio. UV-Vis spectrophotometry was conducted at 1 mM concentrations. TEM (Phillips FEI Tecnai F30 at 3000 keV) was performed to confirm the crystalline nature of each CNP formulation and to determine particle size and morphology.

### Biosensor Fabrication

Si wafers with a natural oxide layer were cut to ~1 × 1 cm squares and cleaned by ultra-sonicating in acetone, ethanol and then water; finally, all wafers were dried under nitrogen. (Synthesis of glassy carbon electrodes detailed in supplementary information as Supplementary method S1) Glassy carbon substrates were then heated to ~300 C and 200 μL of CNPs were dropcast from a 0.2 mM stock solution of CNP3. Following this, the wafers were heated at 300 °C, for 3 hours, in air. Electrodes were subsequently allowed to slowly cool to room temperature inside the furnace.

Electrical contacts were made between glassy carbon (devoid of CNPs) and a polished copper wire using a simple silver paste. The electrodes were then rinsed with de-ionized water and allowed to dry in ambient conditions. When not in use, the electrodes were stored in clear, plastic boxes at room temperature. Electrodes used in blood serum solution were further modified with a thin layer (10 μL of 0.01% in water) of Nafion and allowed to dry overnight under vacuum to prevent protein adsorption to the CNP layer during testing in serum.

### Electrochemical Testing

All potentials were referenced against Ag/AgCl electrode. Prior to any experimental measures, Pt-mesh counter electrodes were electrochemically cleaned by cyclic voltammetry (CV) in 0.5 M H_2_SO_4_ and the potential range was swept between 1.6 and −0.5 V for 50 cycles at 1 V/s. Electrodes were considered clean when CV scans were consistent. Round-disk Au working electrodes (CH instruments) were cleaned by the same method with the potential swept between 1.2 and −0.4 V. The area under the peak corresponding to Au reduction (~0.8 V) was used to represent the electro-active surface area of the working electrode before experimental measurements. Measured current was then converted to current density using the electrochemically active surface area for comparison across multiple measurements.

Initially, CV was performed with CNPs in solution to characterize CNP electrochemical response in presence of hydrogen peroxide. In solution, redox occurs as particles collide with the working electrode surface. Many electrochemical studies involving nanoparticle collisions with electrode surfaces utilize microelectrodes to record individual particle collisions (primarily to determine particle size, shape, and/or composition). For our study, we resolved to determine the electrochemical activity of similar (size, shape, zeta potential) particles. Due to this, large magnitude particle flux at the electrode-solution interface is desirable. This was accomplished using a macro- working electrode for measurements. Consequently, a stepped current response characteristic of high particle flux was observed compared to the spike response observed in the case of microelectrodes (the sustained current response being due to higher [CNP] and greater electrode surface area)^47, 51^. Experimental CV was performed with 1 mM CNPs (each with the three different CNP formulations) dispersed in 10 mL of dH_2_O and a potential range of 0.8 V to −0.3 V at a scan rate of 20 mV/s. H_2_O_2_ was added at 0.01, 0.1, 1, 2.5, and 5 mM concentrations and measurements were repeated five times at each concentration. Electrodes were cleaned as described between each measurement. CV was performed in absence of CNPs as a control. Chronoamperometry (CA) was conducted at −0.23 V, based on the redox activity implicated in CV scans, using 1 mM CNPs 1 and 3. Following the start of a measurement, the current was allowed to equilibrate for 30 s. Then, measurement was run for 10 minutes, with same electrode setup as for CV measurements, with addition of analytes or interfering species (200 μM glucose, sodium nitrite, and uric acid) added by pipette in 100 μL volume increments. Subsequently, thin film CNP- or ceria microparticle -based sensors were fabricated on in-house produced glassy carbon electrodes and tested under identical conditions, without interfering species, to assess the influence of nanoscale material properties in the electrochemical response. Biosensor was evaluated using chronoamperometry performed at the same voltage as for CNPs in solution. Addition of H_2_O_2_ was done in 100 μL increments following an electrochemical equilibration period of 30 s in either 100 mM NaNO_3_ or sheep’s blood serum.

## Conclusions

In the presented study, several formulations of ceria nanoparticles (CNPs) were prepared and tested for electrochemical response in presence of H_2_O_2_. We have shown that the trend observed in CNPs’ Ce^3+^:Ce^4+^ for catalase-mimetic degradation of H_2_O_2_ extends to electrochemical activity; specifically, particles possessing higher Ce^4+^ relative to Ce^3+^ produce superior current response in presence of H_2_O_2_ as determined through cyclic voltammetry and chronoamperometry. This chemical property was then used to fabricate an electrochemical biosensor sensing up to 0.1 pM. Temperature and pH showed no significant effect on sensor sensitivity (high sensor stability) and addition of common interfering species elicited no significant current response (high selectivity). Comparing with other inorganic material-based biosensors, our sensor was capable of detection three orders of magnitude below the most sensitive device in literature. Additionally, the ceria-based biosensor demonstrated excellent H_2_O_2_ detection in blood serum, with a response time of 6 s and a change in sensitivity from 0.156, in buffer solution, to 0.103 μA/log(M)*cm^2^, in blood serum. The low limit of quantitation for H_2_O_2_ detection in blood serum along with physicochemical stability suggests that the sensor is suitable for immediate detection of disease state as part of an implantable device. However, the performance of the biosensor will need to be assessed following incubation in bio-fluid over longer periods of time.

## Electronic supplementary material

Revised Supplementary Information
