# Supplementary material for: Picomolar Detection of Hydrogen Peroxide using Enzyme-free Inorganic Nanoparticle-based Sensor
Source: Sci Rep. 2017 May 2;7:1324. doi: 10.1038/s41598-017-01356-5 (PMC5431009; doi:10.1038/s41598-017-01356-5)
Supplement: Supplementary file 1 — Revised Supplementary Information [file 41598_2017_1356_MOESM1_ESM.pdf]

Picomolar Detection of Hydrogen Peroxide using Enzyme-free Inorganic Nanoparticle-based Sensors

*Craig J. Neal,[a] Ankur Gupta, [a] Swetha Barkam,[a] Shashank Saraf, [a]Soumen Das, [a,b]Hyoung J. Cho, [c] Sudipta Seal[a,b,d]**

[a] *Advanced Materials Processing and Analysis Center*
Materials Science and Engineering, University of Central Florida
4000 Central Florida Blvd, Orlando, FL 32816 (USA)
E-mail: sudipta.seal@ucf.edu

[b] *Nanoscience Technology Center, University of Central Florida*
4000 Central Florida Blvd, Orlando, FL 32816 (USA)

[c] *Mechanical and Aeroscpace Engineering, University of Central Florida*
4000 Central Florida Blvd, Orlando, FL 32816 (USA)

[d] *College of Medicine, University of Central Florida*

*Correspondence to Dr. Sudipta Seal
University of Central Florida
Sudipta.Seal@ucf.edu

S1. *Synthesis of Thin Film Glassy Carbon electrodes.* Thin film glassy carbon electrodes were derived from poly(acrylonitrile) (PAN) polymer. PAN (150,000MW) was dissolved in 8wt% dimethylformamide at 40ºC and maintained at this temperature for 30 min. This solution was spin-coated on Si wafer in two steps. First, 50µL of the solution was dropcast at 2000rpm for 1 min. Then the spin-coating speed was increased to 3000rpm for 30 sec to obtain an even thickness of the PAN coating. The spin-coated Si wafers were then held at 250ºC for 1hr and subsequently placed in a furnace for carbonization under an inert atmosphere (Ar; flow rate: 150mL/min). Samples were then heated to 900ºC at a constant rate of 5Cº/min and held there for 1hr. The samples were then cooled to RT at a rate of 5Cº/min.

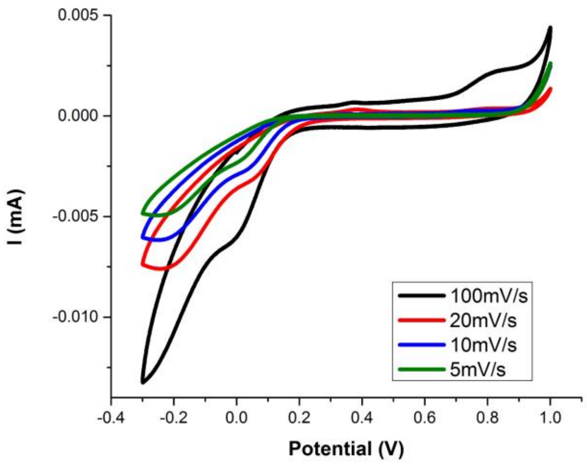

S2. *Current Dependence on Scan Rate for CNP-Hydrogen Peroxide reaction at Reduction peak*. Linear fitting of the peak reduction current vs. the inverse of scan rate produces an R^2 value of 0.99. This is evidence that the CNP-H_2O_2 reaction is diffusion limited at -0.23V.

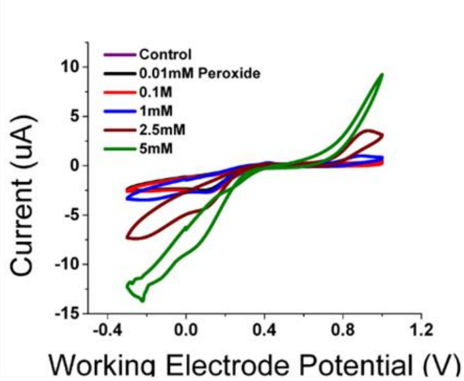

S3. *Peak 2 in CV curves for CNP1 and 2 formulations.* Peak 2 is well-defined and correlates positively with the current density for peroxide reduction at constant peroxide concentration suggesting that hydroxyl species formation is part of the CNP-peroxide reduction mechanism. Corroborating this, when another CNP preparation involving a chloride salt pre-cursor does not show this peak at all as a result of strong, preferential Cl^- adsorption at the working electrode surface [M. Gerlache, Z. Senturk, G. Quarin and J.-M. Kauffmann, *Electroanalysis* 1997, *9*, 1088-1092.]. Interestingly, for CNP3, an anodic current begins at 0.4V in the forward scan, however, the peak is considerably more diffuse than is common for the hydroxyl oxidation. Comparing the scan in Figure 2b. with scans of lower concentration hydrogen peroxide (above), we see that the diffuse peak arises from the convergence of peaks at 0.4 and ~0.7V (this anodic current can thus be decoupled from any background current). This further suggests that lower Ce^{3+}:Ce^{4+} ratio formulations undergo multiple redox reactions involving several oxygen species (based on the substantial peak width and the presence of multiple peaks at lower peroxide concentrations). The width of this peak in Figure 2b. also suggests a capacitive nature for the species involved (charge development at the working electrode surface from adsorbed oxygen species), further inculpating adsorbed oxygen species. Further, it was concluded in the previously referred study that hydrogen peroxide is more easily oxidized at oxidized gold surfaces. It was speculated that hydroxyl species at the electrode surface form hydrogen bonds with peroxides allowing for oxidation at lower potentials [M. Gerlache, 1997], in explanation of the convergence of the multiple peaks at low peroxide concentrations to one common peak partway between the constituent peaks.

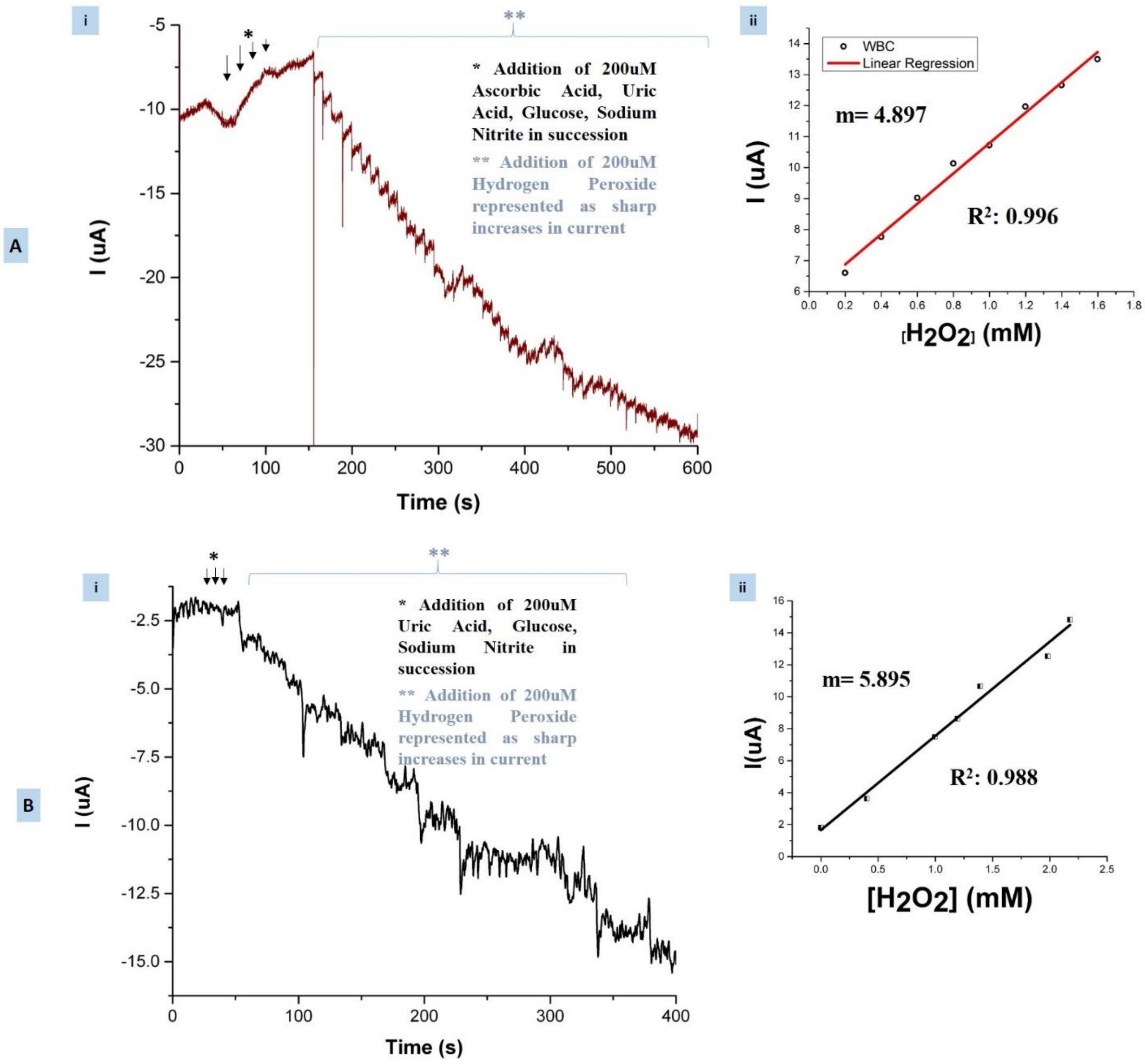

S4. *CA (i) of CNPs1* ***(a)*** *& 3* ***(b)*** *in solution with regression for each* ***(ii)***. **(ai & bi)** Addition of 200uM (final concentrations) of uric acid, glucose, and sodium nitrite in succession shows no significant response for either solution. Addition of 10uL of 200mM H_2O_2 shows instantaneous amperometric response. (**aii and bii**) Regressions for both formulations show high correlation. Slopes of these lines represent sensitivity of detection with CNP3 having greater sensitivity. Additionally, with similar concentrations and addition protocols for hydrogen peroxide, varying the concentration of CNPs in solution predictably changed the current output at peak 1 (Data not shown). Redox active species do not produce measurable current at the working electrode in absence of CNPs, interfering species do not interact with hydrogen peroxide to produce CNP-sensitive by-products, and interfering species do not produce a redox reaction with CNPs which could have produced the observed current.

S5. *Interaction between CNP-coated glassy carbon electrode and ascorbate.* Addition of the electrochemical interferent ascorbic acid produced a small current response for the biosensor without Nafion-coating. The reaction between ceria and ascorbate is established in literature [Sharpe, E., Frasco, T., Andreescu, D. & Andreescu, S. Portable ceria nanoparticle-based assay for rapid detection of food antioxidants (NanoCerac). *Analyst* **138**, 249-262 (2013)]. Interaction likely occurs via the hydroxyl groups of the ascorbate molecule: similar to other studied interactions (e.g. dopamine). Following the application of a thin layer of Nafion, acsorbate addition to test solutions produced no electrochemical response. Pores formed by Nafion naturally conduct protons, but do not allow penetration by electrons nor anions. It is possible that the neutral hydrogen peroxide molecule is able to penetrate the membrane, while ascorbic acid (pka: 4.2) has a negative charge at neutral solution pH's.

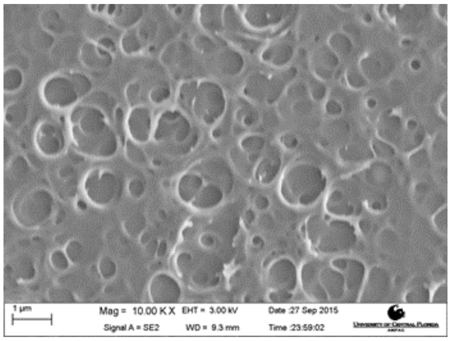

S6. *SEM image of Porous, Glassy Carbon electrode surface.* Porous structure occurring from the thermal degradation of acryl- groups. The pore size shows a bimodal distribution with averages of ~500 and 850nm; ostensibly, facilitating the retention of CNPs on the electrode.
